# Highly Efficient Coupling of Nanolight Emitters to a Ultra-Wide Tunable Nanofibre Cavity

**DOI:** 10.1038/srep09619

**Published:** 2015-05-06

**Authors:** Andreas W. Schell, Hideaki Takashima, Shunya Kamioka, Yasuko Oe, Masazumi Fujiwara, Oliver Benson, Shigeki Takeuchi

**Affiliations:** 1 Department of Electronic Science and Engineering, Kyoto University, Kyoto daigaku-katsura, Nishikyo-ku, Kyoto, Japan; 2 Nano-Optics, Institute of Physics, Humboldt-Universität zu Berlin, Newtonstraße 15, Berlin, Germany; 3 Research Institute for Electronic Science, Hokkaido University, Sapporo, Hokkaido, Japan; 4 The Institute of Scientific and Industrial Research, Osaka University, Mihogaoka 8-1, Ibaraki, Osaka, Japan

## Abstract

Solid-state microcavities combining ultra-small mode volume, wide-range resonance frequency tuning, as well as lossless coupling to a single mode fibre are integral tools for nanophotonics and quantum networks. We developed an integrated system providing all of these three indispensable properties. It consists of a nanofibre Bragg cavity (NFBC) with the mode volume of under 1 μm^3^ and repeatable tuning capability over more than 20 nm at visible wavelengths. In order to demonstrate quantum light-matter interaction, we establish coupling of quantum dots to our tunable NFBC and achieve an emission enhancement by a factor of 2.7.

Solid-state devices that confine light to a very small volume are important to control the optical properties of light emitters. Such devices are especially useful as single photon sources^3^, photonic quantum memories^4^, and photonic quantum gates^5^ in photonic quantum networks^1^,^2^. In those applications, there are three key requirements: an ultra-small mode volume, ideally on the order of *λ*^3^ (where *λ* is the emission wavelength), a wide tuning range of the resonance frequency, and lossless coupling of photons to a single mode optical fibre (SMF). Here, we report on the development of an integrated fibre-coupled all-solid-state device that fulfils all three requirements. First, we fabricate a nanofibre Bragg cavity (NFBC)^6^,^7^ with a diameter of a few hundred nanometres and a mode volume of less than 1 μm^3^. Second, we demonstrate a repeatable tuning of its resonance frequency over an ultra-wide range of more than 20 nm at a visible wavelength (~640 nm), which is possible even at cryogenic temperatures (~85 K). Third, we succeeded in efficiently coupling colloidal quantum dots (QDs) to a NFBC and observed emission enhancement by a factor of 2.7 due to the NFBC. NFBCs with broad tuning capability are not only be expected to be key tools for the realization of nanophotonic quantum networks^1^, but also for numerous real-world applications in photonics, including fibre-embedded zero-threshold microlasers^8^, ultra-wide tunable nanofibre-filters, and ultra-sensitive nanosensors for life sciences^9^.

Solid-state microcavities with small mode volume^10^ come in different types: One- and two-dimensional photonic crystal microcavities^11^,^12^ have recently been used in highly efficient single photon sources^13^ and all-optical switching by single photons^14^. However, for these devices types, the coupling loss of photons to SMFs, which are crucial for most applications, especially in nanophotonic quantum networks, remains an important problem to solve. Alternatively, microspheres^15^,^16^,^17^ and microtoroids^18^ have high quality factors (Q-factors) and can be coupled to SMFs with small coupling loss using tapered optical fibres^15^. However, their mode volumes are large (typically hundreds times of *λ*^3^) and precise coupling via tapered fibres is a demanding task. Furthermore, the tuning range of all these devices usually is limited to about a few nanometres for visible wavelengths and requires changes of experimental conditions such as temperatures^19^,^20^ or the surface laminating layer of the cavity^21^,^22^,^23^.

Tapered optical fibres are increasingly attracting attention as means to couple photons from light emitters to SMFs. When the diameter of the tapered region is about *λ*/2, the field intensity of the propagating mode becomes strong even outside the fibre. This permits the emission mode from the light emitter on the tapered region's surface to efficiently couple to the propagating mode^24^. Since in this way the light is emitted directly in the mode of the SMF, no photons are lost due to additional fibre-coupling or due to small numerical apertures of the collection optics, as they occur for example in microscope systems. Efficient coupling of photons from colloidal QDs^25^,^26^,^27^ and nitrogen vacancy centres in nanodiamonds^28^,^29^ has also been demonstrated. Recently, NFBC devices, where the microcavity is fabricated in the tapered region of the tapered fibre has been proposed by us^7^ and Hakuta^6^ independently, and realization of such devices using ion-beam milling has been reported^30^. Very recently, coupling of an emitter to a cavity formed by an external grating for modulation the refractive indes has been reported^31^.

In this letter, we report on the ultra-wide and repeatable tuning of an NFBC device. We have found that simply by controlling the tension applied to the NFBC, it is possible to tune the resonance frequency by more than 20 nm at visible wavelengths. Furthermore, we have succeeded in coupling colloidal QDs to a tunable NFBC and have observed enhanced emission from the hybrid device.

## Results

**Figure 1:**
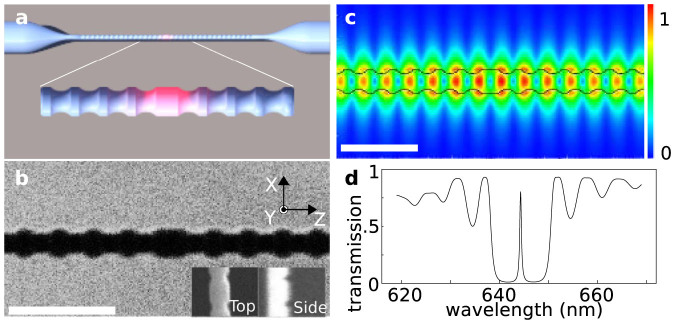
Nanofibre Bragg cavities. (a), Sketch of an NFBC. Note that the number of grating periods has been reduced. (b), SIM image of an NFBC (diameter 270 nm, groove depth 45 nm, pitch 300 nm, and defect length 450 nm). Scalebar is 1 μm. (c), distribution of the electric field calculated using a 3D finite-difference time-domain (FDTD) algorithm. The black line indicates the cavity structure and the scalebar is 1 μm. (d), Transmission spectrum of the NFBC as obtained from the FDTD calculations.

Our NFBCs are fabricated using a focused ion beam (FIB) milling system (see Methods). Typical nanofibre diameters are around 300 nm, which enables single mode operation and efficient coupling to emitters at our target wavelength of 630 nm^25^. Figure 1a shows a schematics of an NFBC, and Figure 1b shows a scanning ion microscope (SIM) image of a fabricated device (3*λ*/4 defect in a 160 period Bragg grating). The depth of the groove, the length of the pitch, and the length of the defect are 45 nm, 300 nm, and 450 nm, respectively.

**Figure 2:**
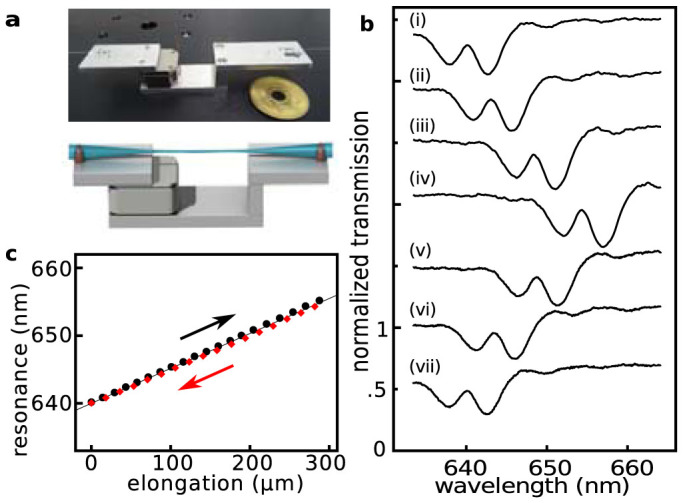
Tuning of the fibre cavities. (a), A schematic diagram and a picture of holder used for tensioning the fibre. (b), Normalized transmission spectra of an NFBC at different tunings. The distances the piezo actuator is moved are 0 μm, 57.5 μm, 160 μm, 270 μm, 176.7 μm, 70.8 μm, and 0 μm for i-vii, respectively. (c), Reversible tuning of an NFBC. The behaviour of the resonance peak when the NFBC is tuned is shown. Black circles indicate stretching of the fibre while red diamonds indicate compressing.

Numerical calculations indicate that this structure yields a mode volume as low as 0.7 μm^3^ and that a large coupling efficiency of over 0.8 can be achieved (see Methods and Supporting Information)^32^. The corresponding electric field distribution is shown in Figure 1c while Figure 1d shows the calculated transmission spectrum. For the cavity mode that appears in the middle of the Bragg gratings' stop band (linewidth of about 10 nm) as a narrow band peak, a numerical Q-factor of about 1600 is found. Measured transmission spectra of an NFBC are shown in Figure 2b. Here, due to imperfections probably caused by drifts during FIB fabrication, the achieved Q-factors are reduced to a value of about 250.

In order to tune the resonance wavelength of the cavities, mechanical tension along the fibre's axis can be applied using a one-axis piezo actuator (see Figure 2a). This allows the resonace to be shifted in a very controlled way, as shown in Figure 2b,c. The slope of the tuning curve is 0.05 nm/μm, which means that an easily controllable step of 1 nm results in a wavelength shift of only 37 MHz. Resonance shifts of up to 25.8 nm can be achieved before the nanofibre ruptures (see Supporting Information). The hysteresis of the resonance on tuning is shown in Figure 2c. The resonance shows linear and reversible behaviour in the wavelength range of over 15 nm. Notably, transmittance and cavity Q-factor are almost constant when tuning the NFBC (variance in Q-factor and transmittance are 4% and 0.7%, respectively). As shown in the Supporting Information, tuning can also be performed at cryogenic temperatures.

Next, we couple light emitters to our NFBCs. For this task, random approaches as for example described in reference^25^ are not suitable since the emitter has to be placed exactly at the cavity's position – in our case a target area just 450 nm in length. Hence, we developed the following approach: A sharp tungsten tip is coated with particles and brought into contact with the nanofibre. Using an optical microscope and an alignment laser coupled to the fibre, we ensure that the tip only touches the fibre at the cavity's position. On retraction of the tip, it is highly probable that only one, or a few, particles are deposited. In the following, we show coupling of QDs (emission wavelength 630 nm) to the NFBCs.

**Figure 3:**
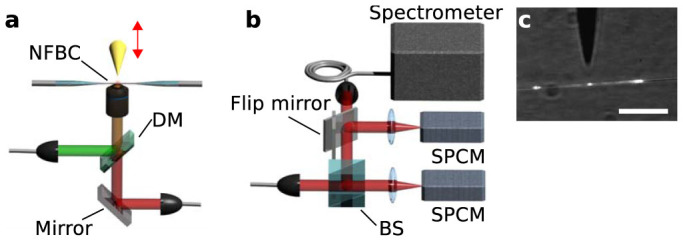
Experimental setup. (a), Sketch of the microscope setup. An NFBC is placed in front of a microscope objective, which is used either for fibre coupled confocal microscopy or wide field imaging. A sharp tip is used to place nanoparticles on the fibre in a controlled way. DM is a dichroic mirror. BS is a beam splitter. (b), Sketch of the fibre coupled detection system used. Two single photon counting modules (SPCM) in Hanbury Brown and Twiss (HBT) configuration are used to measure the photon stream. One arm of the HBT setup can also be coupled into a spectrometer. (c), Camera image of the coupling process. Scalebar is 20 μm.

**Figure 4:**
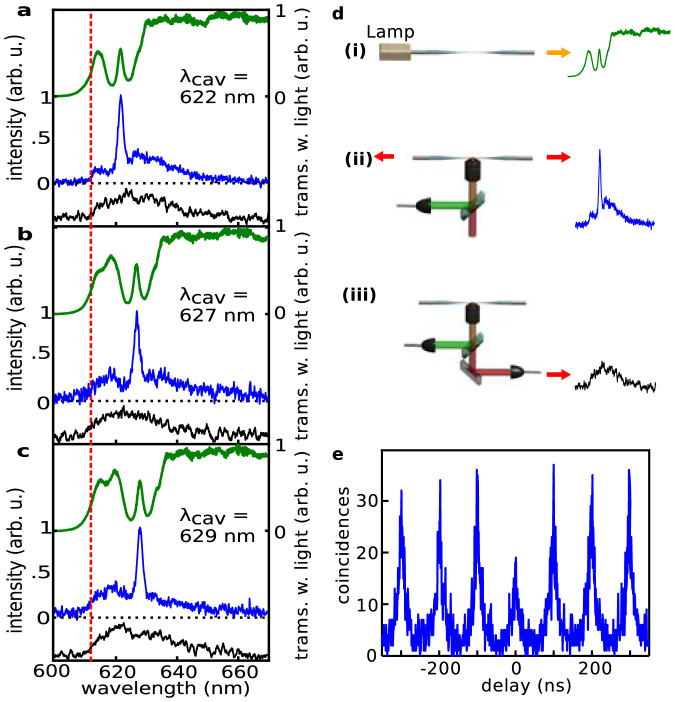
Fluorescence spectra of a few QDs coupled to a nanofibre cavity. (a), (b), (c), Measured signal for three different tunings (622 nm, 627 nm, and 629 nm) of the fibre nanocavity. Spectra acquired using a halogen lamp are shown in green (i in panel (d)), spectra acquired through the fibre while exciting using the microscope objective are shown in blue (ii in panel (d)), and spectra acquired using the confocal microscope are shown in back (iii in panel (d)). The dashed vertical line indicates the cut-on wavelength of the longpass filters used and the black curves are offset by −0.2 arb u for clarity. (d), Sketch of the different setups used. (e), Antibunching measurement in confocal configuration under pulsed excitation. From the clear dip going below 0.5 it can be concluded that the main contribution to the signal stems from a single emitter.

The experimental setup used to investigate coupling of emitters to our NFBCs (see Figure 3) consists of a microscope with a three-axis translation stage and a tensionable fibre mount. Light sources (lasers, halogen lamp) and detection units (spectrometer, avalanche photodiodes in Hanbury Brown and Twiss configuration) are all fibre coupled making it easily possible to attach the sources/detectors either to the ends of the tapered fibre or to the microscope. Figure 4 shows measurements of a few QDs coupled to an NFBC. Three different experimental configurations (see Figure 4d) are used. In the first, a laser of 532 nm wavelength is focussed via the microscope objective and fluorescence is collected using the same objective in a confocal arrangement (black curves in Figure 4a–c). In the second configuration, light from a halogen lamp is coupled into the fibre and transmitted light is measured (green curves in Figure 4a–c). Finally, in the third configuration, the emitters are again excited by the laser through the objective lens, and the light from both fibre ends is measured (blue curves in Figure 4a–c). In Figure 4 as well as in the following analysis, averaging over both fibre ends is performed in order to exclude effects stemming from a potential asymmetry of NFBC or fibre. While the confocally detected spectrum exhibits a Gaussian shape, the light detected at the fibre's ends clearly shows an enhancement at the cavity's resonance and suppression in the stop band of the Bragg gratings. When the NFBC is tuned, the enhanced emission clearly shifts with the cavity resonance. Note that in contrast to temperature tuning or gas condensation tuning via tension leaves the emitter unaffected. This is a clear indication that the cavity is the sole source of the fluorescence enhancement. An evaluation of the fluorescence maxima detected through the fibre and comparison with the confocal measurement yields enhancement factors of approximately 2.7 for all three cavity tunings shown, which is in excellent agreement with theoretical predictions. More specifically, when taking into account the measured Q-factor of this NFBG (*Q* = 330) and the mode volume of 0.7 μm^3^ derived from the FDTD calculations, an enhancement factor of approximately 3 is expected when the Purcell formula is used (Supporting information). Using improved structures with Q-factors closer to the calculated value, this enhancement could even reach 14. Note that this is a first implementation and it is highly probable that significant improvements to this value can be achieved by more sophisticated designs and more accurate fabrication, as it was done with two-dimensional photonic crystal and nanobeam cavities.

## Conclusion

In conclusion, we have introduced the system of nanofibre Bragg cavities with small mode volumes on the order of a cubic wavelength and an ultra-wide tuning range. The NFBGs are intrinsically fibre coupled, which, together with their high quality factors and small mode volumes, makes them ideally suited for use in altering the photonic properties of quantum emitters. A hysteresis-free tuning with a range of more than 20 nm, which is achieved simply by straining the NFBC, enables adjustment of cavity-emitter coupling without changing other experimental parameters such as temperature or surface layers. The NFBCs can be operated and tuned under cryogenic conditions, making it feasible to couple them with emitters that need cryogenic temperatures. We have also shown controlled coupling of QDs to NFBCs resulting in an enhancement of their emission by a factor of 3 – an enhancement that probably can be increased by more advanced design and fabrication techniques. Note that, in principle, other particles, e.g. nanodiamonds containing defect colour centres^29^,^33^, can also be coupled to our NFBC using the technique introduced here. With these properties, NFBCs will improve single photon sources for nanophotonic quantum networks and will enable efficient implementation of important devices needed for quantum information science, such as single photon switches.

## Methods

### Fabrication of nanofibre cavities

A focused ion beam milling system (SMI-2050, Seiko Instruments Inc.) is used to etch the cavity structures. The nanofibres are fabricated by heating a single-mode optical fibre with a ceramic heater and stretching it into a fine thread. Ga^+^ ions of 30 kV accelerating voltage and 9.3 pA beam current are focused on the nanofibre with the spot size of 13 nm and scanned perpendicularly to the direction of the long axis of the fibre in order to fabricate a 160 periods grating with one defect in the middle.

### Numerical calculations

In order to analyse the fibre cavities, 3D finite-difference time-domain (FDTD) simulations are performed using a commercial package (FDTD Solutions, Lumerical). The modelling geometry, which is simplified from the structure utilized in the experiment, can be found in the Supporting Information. Grooves with a period of 300 nm and a depth of 45 nm are carved on the upside and transverse sides of the nanofibres (diameter 300 nm). A 450 nm defect is introduced in the centre of the 160 period grating. The simulation region is 60 × 2 × 2 *μ*m and perfectly matched layers (PML) are employed as absorbing boundary conditions. Time steps and simulation time are 0.039 fs and 5 ps, respectively. We use an automatic non-uniform mesh with a high accuracy and the material properties of SiO_2_ provided by Lumerical in the calculation. The light source is placed at a location −24.5 μm away from the centre of the simulation region and excited the fundamental mode of the nanofibre. The transmittance is monitored at 25 μm away from the centre of the simulation region.

### Emitter-cavity coupling

For coupling emitters and cavities colloidal quantum dots (QSP630, Ocean Nano Tech) dispersed in toulene are used. A sharp tungsten tips (TP-0002, Micro Support) is dipped in the QD solution. An appropriate concentration of QDs in toluene (approx. 0.06 mg/ml) is used to ensure that only a few QDs stick to the tip's apex. Using a three axis translation stage equipped with stepper motors and piezo actuators, the tip's apex is brought in contact with the cavity and retracted subsequently. The process is monitored using the microscope described below and an off-resonant laser is coupled to the fibre to provide additional optical feedback via scattering of the evanescent field due to the tip.

### Optical measurements

The microscope used is an IX 71 (Olympus) with an high numerical aperture objective (MPLAPON 100×/0.95). Optical images are acquired using a CCD camera (PRO EM 512 B, Princeton instruments). During confocal operation, a laser beam is sent through the back port and the emitted fluorescence light is coupled in a multimode fibre (P1-1550A-FC-2, Thorlabs) using a fibre coupler intalled after the tube lens. Spectra are acquired using a spectrometer (MS257, ORIEL Instruments) with a CCD camera (DU420-OE, Andor).

Spectra in Figure 2 are normalized to the transmission of a non-tapered single mode fibre and the blue curves in Figure 4 are averages of both fibre ends. The avalanche photodiodes used in the HBT are SPCM (Perkin Elmer).

## Supplementary Material

Supplementary Information Supporting Information
