# Supplementary material for: Highly Efficient Coupling of Nanolight Emitters to a Ultra-Wide Tunable Nanofibre Cavity
Source: Sci Rep. 2015 May 6;5:9619. doi: 10.1038/srep09619 (PMC5386186; doi:10.1038/srep09619)
Supplement: Supplementary Information — Supporting Information [file srep09619-s1.pdf]

# Highly Efficient Coupling of Nanolight Emitters to a Ultra-Wide Tunable Nanofibre Cavity: Supporting Information

Andreas W. Schell,[*,†,¶,§,‖] Hideaki Takashima,[†,¶,§,‖] Shunya Kamioka,[¶,§,‖] Yasuko Oe,[†,¶,§] Masazumi Fujiwara,[‡,¶,§] Oliver Benson,[‡] and Shigeki Takeuchi[*,†,¶,§]

*Department of Electronic Science and Engineering, Kyoto University, Kyoto daigaku-katsura, Nishikyo-ku, Kyoto, Japan, Nano-Optics, Institute of Physics, Humboldt-Universität zu Berlin, Newtonstraße 15, Berlin, Germany, Research Institute for Electronic Science, Hokkaido University, Sapporo, Hokkaido, Japan, and The Institute of Scientific and Industrial Research, Osaka University, Mihogaoka 8-1, Ibaraki, Osaka, Japan*

E-mail: andreas.schell@physik.hu-berlin.de; takeuchi@kuee.kyoto-u.ac.jp

## Nanofibre Bragg cavities

Nanofibre Bragg cavities (NFBCs) are created by tapering down a standard optical fibre (630 HP, Thorlabs) using a homebuilt tapering machine to a diameter of approximately 300 nm and then using a focused ion beam to mill periodic groves into the tapered region. The

[*]To whom correspondence should be addressed

[†]Department of Electronic Science and Engineering, Kyoto University, Kyoto daigaku-katsura, Nishikyo-ku, Kyoto, Japan

[‡]Nano-Optics, Institute of Physics, Humboldt-Universität zu Berlin, Newtonstraße 15, Berlin, Germany

[¶]Research Institute for Electronic Science, Hokkaido University, Sapporo, Hokkaido, Japan

[§]The Institute of Scientific and Industrial Research, Osaka University, Mihogaoka 8-1, Ibaraki, Osaka, Japan

[‖]These authors contributed equally.

grooves are milled from one side, resulting in an arch-like shape. Their depth is 45 nm. To yield a Bragg grating with a design wavelength of 630 nm, the spatial period of the grooves $\Lambda_B$ is adjusted according to the Bragg formula:

$$\lambda_B = 2n_{eff}\Lambda_B, \tag{1}$$

where $n_{eff}$ is the effective refractive index of the nanofibre. Putting in the calculated effective index $n_{eff}$=*1.07* for the wavelength of 630 nm and the fibre diameter of 300 nm, the grating period $\Lambda_B$ is determined to be 300 nm. The grooves achieve a large refractive index modulation $\Delta n$ of $\sim$ 0.066 as calculated via finite difference time domain simulations. In the middle of the Bragg grating, a defect of $\frac{3}{2}\Lambda_B$ is introduced in order to form the cavity. The resulting structure is shown in Figure 1a,b. Figure 1c,d show the simplification of the NFBC structure we used in the numerical calculations.

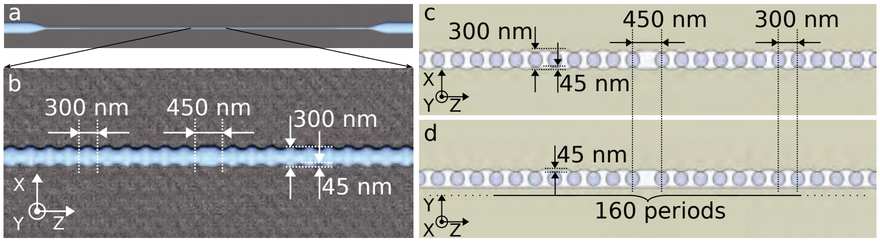


Figure 1: Nanofibre Bragg cavities. **a** and **b** are schematic views of the NFBCs while **c** and **d** show simplified structures as used in the numerical calculations

## Simulation of the coupling efficiency

The coupling efficiencies to a fundamental mode of the fibre are calculated based on our recent paper.[1] Figure 2 shows the geometry used for the simulation. The dipoles are placed at weak and strong points of the electric field inside the cavity. The direction of the dipoles is the X-axis. The wavelength of the dipoles is set to the resonance of the NFBC (634.32 nm). The coupling efficiencies for one end of the fibre are monitored at 28 μm away from the centre of the simulation region and then summed up for the total coupling efficiency.

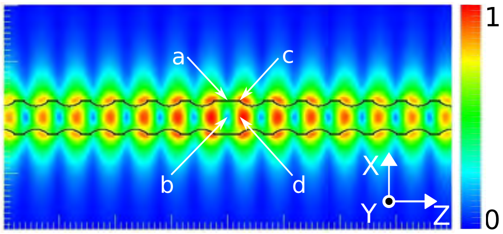


Figure 2: Electric field at a NFBG. A cross-section of the electric field distribution at the centre of the fibre about the Y axis (Y = 0 μm). The black line indicates the cavity structure. The positions of the dipoles (a, b, c and d) for the simulation of the used in the simulations for the coupling efficiency are marked by arrows.

Table 1 shows the calculation results for the cases where a single dipole is located at the corresponding position. When the dipole is placed at positions with a weak electric field (a and b), the coupling efficiency is very low. In contrast, when the dipole is placed at positions with a strong electric field (c and d), the coupling efficiency is over 0.8.

Table 1: Calculation results for the coupling efficiencies for four dipolar positions.

| | X ($\mu$m) | Z ($\mu$m) | Coupling efficiency |
|---|---|---|---|
| a | 0.15 | 0 | 0.009 |
| b | 0 | 0 | 0.013 |
| c | 0.15 | 0.118 | 0.812 |
| d | 0 | 0.118 | 0.838 |

# Purcell formula and broad band emitters

Coupling of emitters to optical cavities results in a coupled system with new properties. In the so called weak coupling limit, where dissipation is dominating, this leads to an enhancement of the emitter's emission rate via the Purcell effect.[2] The emission enhancement factor is given by the Purcell formula:

$$P = \frac{3}{4\pi}\left(\frac{\lambda}{n}\right)^3 \frac{Q}{V}, \tag{2}$$

with $\lambda$ being the free space emission wavelength of the emitter, $n$ the refractive index, $Q$ the quality factor of the cavity, and $V$ its mode volume.

For a broad band emitter, where only parts of the emission spectrum are inside the cavities resonance, the situation is more difficult. Only a part of the decay channels get enhanced by the factor $P$. In the emitters spectrum, the relative strength of the emission at one wavelength $\lambda'$ in a spatial mode $m$ $I_{\lambda',m}$ is:

$$I_{\lambda',m} = \frac{k_{\lambda',m}}{\sum\limits_{\lambda,n} k_{\lambda,n} + k_{nr}}\,, \tag{3}$$

where $k_\lambda, n$ are the decay rates for transitions at the wavelengths $\lambda$ into mode $n$ and $k_{nr}$ is the non radiative decay rate. Sorting the decay rated at different wavelengths and modes into the ones that will be affected by the cavity $k_{cav}$ and the unaffected ones $k_{free}$ results in:

$$I_{cav} = \frac{k_{cav}}{k_{cav} + k_{free} + k_{nr}}\,, \tag{4}$$

where we have introduced $I_{cav}$ as the relative intensity of the part of the spectrum, which will be affected by the cavity. When coupling to the cavity, the rate $k_{cav}$ gets enhanced by the Purcell factor $P$ resulting in an enhanced emission into the cavity $I_{enh}$:

$$I_{enh} = \frac{Pk_{cav}}{Pk_{cav} + k_{free} + k_{nr}}\,. \tag{5}$$

For situations, where decay into other channels dominates over the enhanced decay into the cavity as it is the case for our experiment, the denominator of Equation 5 can be assumed to be constant, leading to a direct connection of Purcell factor and emission enhancement:

$$I_{enh} \approx PI_{cav}\,. \tag{6}$$

## Tuning at cryogenic temperatures

Figure 3a shows our homebuilt cooling system for tuning at cryogenic temperatures. The system consists of a volume thermally isolated from the environment by a polystyrene foam container, in which the translation stage hosting the fibre cavity is fixed on an additional metal case. To monitor the temperature around the sample, a T type thermocouple is fixed next to the stage. For the input and output of light and refrigerant, two feed-throughs are mounted on the walls of the container. Single mode optical fibres are directly installed in one of the container feedthroughs and connected to the fibre cavity using a fibre splicer. To prevent icing on the fibre, evaporated nitrogen gas is employed as the refrigerant. Pure nitrogen gas is cooled with a heat exchanger and flowed through the container. Temperatures reached as low as 85 K in approximately 30 minutes.

Figure 3b shows the resonance shift on tensioning the fibre and the corresponding transmittance of a NFBC at 85 K. Its resonance wavelength is red-shifted linearly in a range of 25.8 nm. The transmittance is almost constant during the tuning. No significant transmittance reduction is observed between the room temperature and 85 K (not shown).

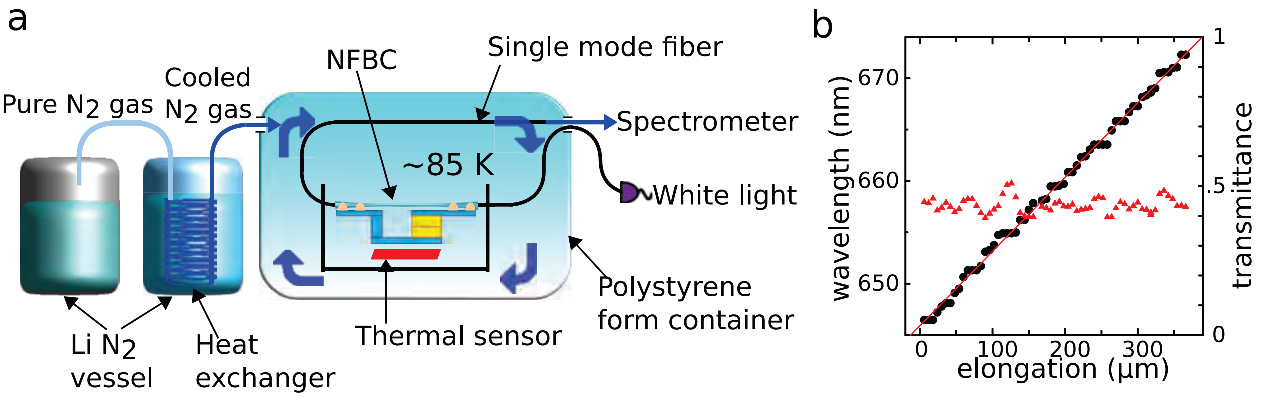


Figure 3: Tuning at cryogenic temperatures. **a** shows a schematic diagram of our home-made cooling system. Pure nitrogen gas is cooled in a heat exchanger and then used to cool down the NFBG, which can be tuned and is connected to a light source and a spectrometer. **b** shows a tuning measurement performed at 85 K. Black dots are the resonance wavelength while the red triangles are the corresponding transmission. The red line is a fit to the data.

# Coupling of QDs to tapered fibre cavity

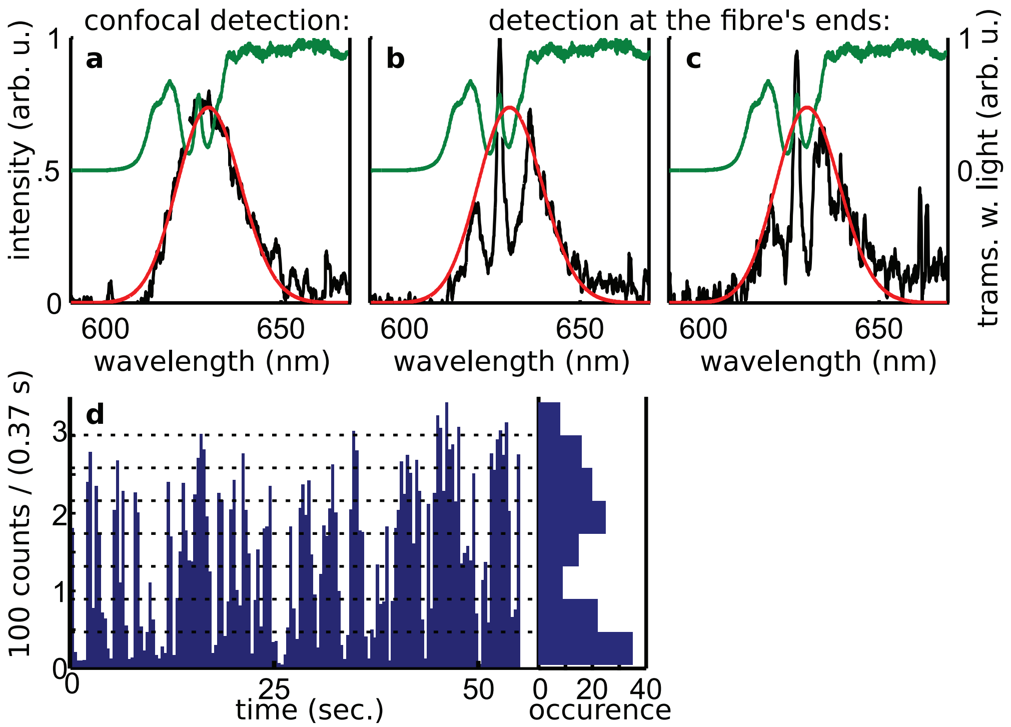


Figure 4: Fluorescence spectra of a single quantum dots (QDs) coupled to a nanofibre cavity. Panels **a,b,c**, show the detected spectra in confocal configuration and at the fibre's ends. The measured data is shown in black and a a Gaussian energy distribution fitted to the confocal measurementsis shown in red. In green the corresponding cavity resonance is shown. An enhancement of the single QD's fluorescence is visible at the resonance wavelength as well as strong suppression in the Bragg mirrors' band gap. This behaviour indicated that the QD's spatial position is not exactly at the cavity, but slightly shifted towards one of the mirrors. In **d** a time trace of the QD's emission as collected through the fibre is shown. A clear two-level blinking indicates the presence of a single quantum emitter.

In order to get to the single-emitter regime – the regime needed, for example, for efficient single photon sources – we bleach the emission from the quantum dots with the excitation laser until the emission is considerably darker. The resulting emission spectrum is shown in Figure 4. While the main features are similar to the case of a few QDs, the enhancement is less pronounced while the emission inhibition inside the band gap is larger. This means that in the bleaching process a QD survived that is not perfectly coupled. On the other hand, this also means that coupling for the now bleached particles was even better than calculated. To prove the single emitter character of the remaining quantum dot, we look at its blinking

behaviour as shown in Figure 4d where two-level blinking is visible – a clear indication for single quantum systems.[3]

## References

(1) Almokhtar, M.; Fujiwara, M.; Takashima, H.; Takeuchi, S. *Opt. Express* **2014**, *22*, 20045–20059.

(2) Vahala, K. J. *Nature* **2003**, *424*, 839–846.

(3) Empedocles, S.; Bawendi, M. *Accounts of chemical research* **1999**, *32*, 389–396.
